# Cadmium Exposure and Noncommunicable Diseases in Environmentally Exposed Brazilian Population: Cross-Sectional Study without Association of *GSTP1* Polymorphism

**DOI:** 10.3390/toxics12090640

**Published:** 2024-08-31

**Authors:** Jamila Alessandra Perini, Yasmin Marinho Henriques da Silva, Mayara Calixto da Silva, Beatriz Pegado Silva, Daniel Escorsim Machado, Maria de Fátima Ramos Moreira

**Affiliations:** 1 Research Laboratory of Pharmaceutical Sciences (LAPESF), Rio de Janeiro State University (West Zone-UERJ-ZO), Rio de Janeiro 23070-200, RJ, Brazil; yasmin.sil.marinho@gmail.com (Y.M.H.d.S.); mayaracx_2010@hotmail.com (M.C.d.S.); beahcaught@gmail.com (B.P.S.); danielescorsim@yahoo.com.br (D.E.M.); 2 Post-Graduation Program in Environmental Science and Technology, Rio de Janeiro State University (West Zone-UERJ-ZO), Rio de Janeiro 23070-200, RJ, Brazil; 3 Center for Studies on Worker Health and Human Ecology (CESTEH), National School of Public Health (ENSP), Oswaldo Cruz Foundation (Fiocruz), Rio de Janeiro 21041-210, RJ, Brazil; fatima.moreira@fiocruz.br

**Keywords:** cadmium, GSTP1, genetic polymorphisms, toxicokinetic, environmental exhibition, industrial waste

## Abstract

Cadmium (Cd) is a toxic metal which is harmful to humans and the environment. Cd levels and adverse effects may be associated with genetic polymorphisms in genes involved in its toxicokinetics. This study investigated Cd levels in 198 residents of a condominium in Rio de Janeiro, Brazil, built on industrial steel slag waste and the influence of glutathione S-transferase pi isoform 1 (*GSTP1*) rs1695 A>G polymorphism. Polymorphism was genotyped using a validated TaqMan assay; Cd levels were measured in blood (BCd) and urine (UCd) by graphite furnace atomic absorption spectrometry. Associations were evaluated by multiple logistic regression, odds ratios (ORs), and 95% confidence intervals (CIs). The mean Cd levels were 0.70 ± 0.20 µg L^−1^ (BCd), 0.58 ± 0.57 µg L^−1^ (UCd), and 0.61 ± 0.65 µg g^−1^ in urine corrected by creatinine (UcCd), and the Cd results were above tolerable levels (BCd > 0.5 µg L^−1^) in 87.4% of subjects. Higher blood Cd levels (>0.69 µg L^−1^) were associated with respiratory disease (OR = 2.4; 95%CI = 1.2–5.0), as almost 30% of people with respiratory diseases had higher Cd levels. The *GSTP1* rs1695AA genotype frequency was 38.1%, and there were no significant differences between the SNP and Cd levels. High Cd levels and a high prevalence of diseases highlight the importance of implementing public policies and the continuous monitoring of this at-risk population.

## 1. Introduction

Cadmium (Cd) is one of the major constituents of waste generated by the steel industry and is continuously released into the environment from natural and anthropogenic sources, primarily from its use in the galvanization of steel but also from the combustion of fossil fuels and the use of cadmium-containing scrap metal [1]. As a result, humans and animals are exposed primarily through the consumption of contaminated food and water, as well as through air pollution, occupational activities, and smoking [2,3]. Long-term environmental exposure to Cd can pose a threat to human health and the environment. Its adverse health effects have been associated with several diseases, including cancer, central nervous system and liver diseases [4], chronic obstructive pulmonary disease (COPD) [5,6], reproductive and cardiovascular diseases, placental lesions and endocrine disruption [7], chronic kidney disease [8], and musculoskeletal and neurodegenerative diseases [4]. Cd has therefore been classified as a human carcinogen by the International Agency for Research on Cancer (IARC) [9,10].

The main routes of exposure to Cd are inhalation and ingestion, with approximately 25 to 60% of the exposed dose being absorbed through the respiratory tract. Smokers and occupationally exposed individuals have higher risks of absorption. Once absorbed, Cd is distributed through the blood to various organs, primarily the kidneys and the gastrointestinal tract (GIT). The estimated biological half-life of Cd in the GIT is 4 to 19 years, while in the kidney, it is 6 to 38 years. Cd is primarily absorbed by the kidneys, and the main excretion route is renal. However, 2–8% of the dose is absorbed by the GIT, and specifically in the liver, it is bound to glutathione (GSH) and excreted in bile. Individual susceptibility to Cd exposure levels is likely related to genetic variations in enzymes involved in Cd metabolism, such as glutathione S-transferase (GST), which plays a critical role in Cd detoxification by conjugating with GSH, resulting in the excretion of metal conjugates in urine and feces [11]. The main gene involved in the biotransformation and detoxification of Cd is the phase II enzyme GST [12]. Among the different GST isoforms, the pi 1 isoform of glutathione S-transferase (GSTP1) is present in large quantities in the lung and therefore mediates the metabolism and detoxification of inhaled toxic substances [13]. *GSTP1* is a polymorphic gene located on chromosome 11 (11q13.2), and the single-nucleotide polymorphism (SNP) rs1695 313A>G alters enzymatic activity by amino acid exchange (Ile105Val), reducing substrate affinity ~3-fold in vitro [14]. Therefore, this SNP may be associated with increased susceptibility to Cd toxicity. In addition, it has an increased frequency in various populations [15]. In this context, the objectives of this study were to evaluate Cd levels in individuals with chronic environmental exposure to the metal; to describe their sociodemographic characteristics, noncommunicable diseases (NCDs), and frequency of *GSTP1* rs1695 SNP genotypes; and to evaluate the influence of the SNP on Cd levels.

## 2. Materials and Methods

### 2.1. Study Population and Clinical Evaluation

The present cross-sectional study was approved by the Research Ethics Committee of the National School of Public Health (CAAE number 40554820.9.0000.5240) and includes a convenience sample of residents of the Volta Grande IV condominium, located in Volta Redonda, Rio de Janeiro, Brazil. Residents were randomly selected and invited to participate in this study. Inclusion criteria were adults over 18 years of age who had lived in the condominium for at least six months and had the autonomy to answer the questionnaire. Then, the residents were invited to participate in this study, and the final sample consisted of those who accepted the invitation. After signing the free and informed consent form, sociodemographic, environmental, and clinical information was obtained from previously validated questionnaires and from the clinical assessment [1,16]. Clinical assessments were conducted in a single meeting, between April 2017 and October 2019, by appropriately trained professionals, according to routine protocols of the primary health care center. To determine the cadmium exposure levels in the study population, blood and urine samples were collected during the evaluations [16]. Therefore, the present study included 198 individuals for which the following data were available: Cd level, age, and DNA sample for genetic analysis.

### 2.2. Cadmium Determination

The estimates of Cd exposure were measured in blood (BCd) and urine (UCd) samples. All values obtained for UCd were corrected by creatinine (UcCd). Blood samples were collected in metal-free, heparinized vacutainer tubes, while urine was collected directly in polyethylene containers that had been previously tested for Cd contamination. Both samples were transported to the laboratory under refrigeration and kept frozen until analysis. The creatinine concentration in urine was determined with a colorimetric kit (Doles Reagents and Equipment for Laboratories Ltd., Goiânia, GO, Brazil) using the spectrophotometric method with direct measurement by reaction with picric acid in alkaline medium, after deproteinization.

Blood and urine samples were diluted in Triton X-100 and nitric acid, respectively, prior to measurement. Two atomic absorption spectrometers (Analyst 800 and 900, Perkin-Elmer, Shelton, CT, USA) equipped with a transverse electrothermal nebulizer, a longitudinal Zeeman background corrector, an AS-800 autosampler, end-cap pyrolytic graphite tubes, and Lumina hollow cathode lamps, all Perkin-Elmer, determined the concentration of BCd and UCd. The accuracy of the results was monitored by analyzing the following reference materials in each series of samples: Contox Heavy Metal Blood Control and Contox Metal Serum Control (Kaulson Laboratories, West Caldwell, NJ, USA); Lyphochek Urine Metals Control (Bio-Rad, Hercules, CA, USA); and Toxic Metals in Freeze-Dried Urine SRM 2670 (NIST, Gaithersburg, MD, USA).

### 2.3. Polymorphism Genotyping

**Figure 1:**
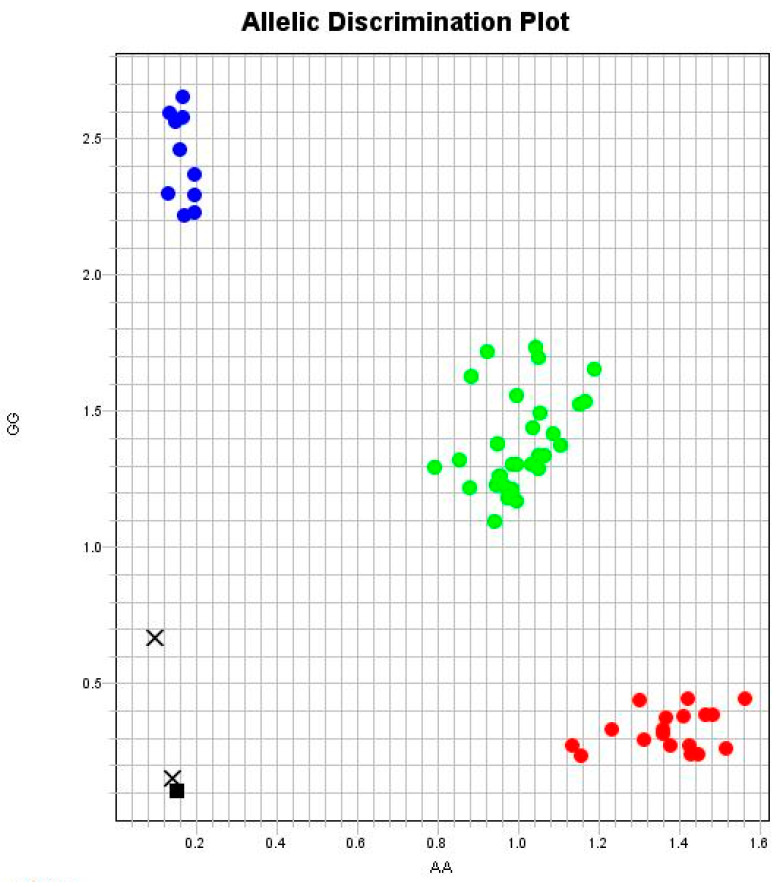
An analysis of the *GSTP1* rs1695 polymorphism by real-time PCR using the TaqMan system. (■) Black squares are negative controls, which should not show PCR amplification. (●) The red circles are individuals with the wild homozygous genotype (*AA*), (●) blue circles are the variant homozygotes (*GG*), and (●) green circles are the heterozygotes (*AG*) showing the fluorescence of both probes. (X) Black Xs are samples with an undetermined genotype.

Genomic DNA was extracted from blood samples using an extraction kit (Qiagen, Hilden, Germany) according to the manufacturer’s recommended procedures. A genotyping analysis of the *GSTP1* rs1695 A>G SNP was performed on the 7500 Real-Time PCR System (Applied Biosystems, Foster City, CA, USA) using a validated TaqMan assay (C_3237198_20) for allelic discrimination, as previously described by our group [17,18]. Each reaction used two standardized positive controls of each polymorphism genotype to ensure genotyping quality. The probes were labeled with different fluorescence, which was captured by the instrument software and discriminated the individual’s genotype *GSTP1* rs1695 *AA*, *AG*, or *GG* (Figure 1).

### 2.4. Statistical Analysis

Continuous variables were presented as the mean ± standard deviation (SD) if they were normally distributed or as the median and range (minimum and maximum) if they were not normally distributed. Linearity was tested using the Shapiro–Wilk test, and associations between continuous variables were assessed using Spearman’s linear correlation coefficient. Categorical variables were described as numbers (n) and percentages (%), and associations between them were analyzed using the chi-squared test or Fisher’s exact test, when appropriate, and the estimation of odds ratios (ORs) and their respective 95% confidence intervals (95% CIs).

Hardy–Weinberg equilibrium (HWE) was assessed by the X2 goodness-of-fit test to investigate perturbations in the genotype and allele frequencies of the *GSTP1 313* A>G SNP. Cadmium levels were also treated as categorical variables according to the median values, and their associations with the *GSTP1 313* A>G SNP were adjusted by variables that had a significance level lower than or equal to 0.20 in the univariate analysis (*p*-value ≤ 0.20) to select variables for adjustment, but they remained with a significance level of 0.05 (*p*-value ≤ 0.05) after model exit, as described elsewhere [16]. All statistical analyses were performed using R software (R Foundation for Statistical Computing, Vienna, Austria, version 4.2.1) with a 5% significance level.

## 3. Results

**Figure 2:**
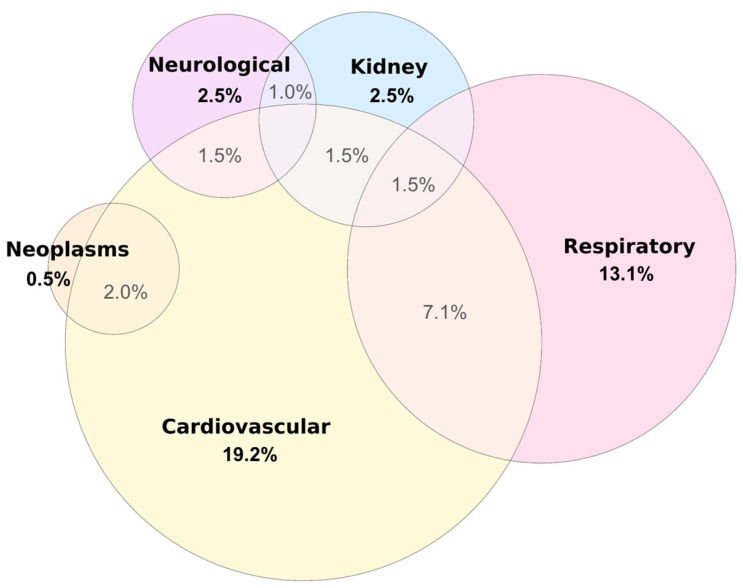
Prevalence of noncommunicable diseases in study participants (*n* = 198). Participants could have more than one NCD: 7.1% of participants had cardiovascular and respiratory diseases; 2.0% had cardiovascular disease and neoplasms; 1.5% had cardiovascular and renal diseases; 1.5% had cardiovascular and neurological diseases; 1.0% had renal and neurological diseases; and 1.5% of participants had respiratory, renal, and cardiovascular diseases.

The present study included 198 individuals who had all three of the following information: Cd level, age, and DNA sample for genetic analysis. Among them, 58% were female, with an average age of 50 ± 14.9 years (ranging from 18 to 86 years), 29.3% were smokers, 48.4% drank alcoholic beverages, and the mean length of residence in the condominium was 15 ± 4.9 years (ranging from 1 to 22 years), with most people living there for at least 15 years (64.8% of those 189 who had this information available). The clinical history revealed a high prevalence of NCDs among the participants of both genders, with only 92 participants (46.5%) having no NCD. Therefore, 53.5% presented at least one of the following NCDs: cardiovascular, respiratory, neurological, or kidney diseases and neoplasms (Figure 2), while 14.1% had two diseases, and 1.5% had three NCDs. In addition, 0.5% of participants had respiratory and neurological diseases, and 0.5% of participants had renal diseases and neoplasms.

Demographic and clinical characteristics were compared between men and women. There was no significant difference between the variables analyzed, except for alcohol consumption: 37.2% of women and 64.6% of men (*p* = 0.001, χ^2^ test).

**Figure 3:**
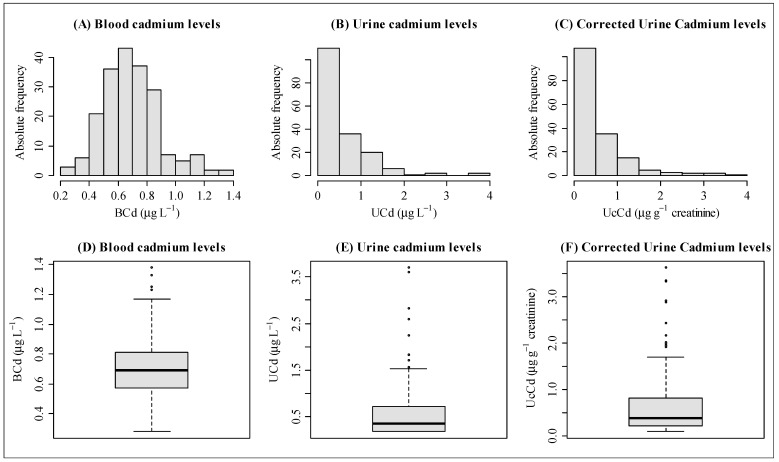
Distribution profile of Cd levels from blood (*n* = 198), urine (*n* = 177), and creatinine-corrected urine samples (*n* = 170). (**A**,**D**) Cd levels in blood (BCd = 0.28–1.38 µg L^−1^); (**B**,**E**) Cd levels in urine (UCd = 0.18–3.70 µg L^−1^); (**C**,**F**) Cd levels in urine corrected by creatinine (UcCd = 0.09–3.63 µg g^−1^ creatinine).

Blood samples were collected from 198 individuals, while only 177 individuals provided urine samples for Cd measurement, and 170 urine samples were corrected by creatinine (Figure 3). None of the three Cd measurements (BCd, UCd, and UcCd) showed normal distributions (Shapiro–Wilk test *p*-value < 0.05) (Figure 3A–C). The mean Cd levels in blood and urine samples were 0.70 ± 0.20 µg L^−1^ (median = 0.69 µg L^−1^, ranging from 0.28 to 1.38 µg L^−1^) and 0.58 ± 0.57 µg L^−1^ (median = 0.36 µg L^−1^, ranging from 0.18 to 3.70 µg L^−1^), respectively, while the mean Cd level in urine after correction for creatinine was 0.61 ± 0.65 µg g^−1^ creatinine (median = 0.38 µg µg g^−1^ creatinine, ranging from 0.09 to 3.63 µg g^−1^ creatinine) (Figure 3D–F). Considering the reference value for Cd in blood of <0.5 µg/L^−1^ for all ages [10], 173 (87.4%) individuals had Cd results above tolerable levels. The levels of Cd in women and men were not significantly different: BCd = 0.70 ± 0.18 µg L^−1^ versus 0.71 ± 0.22 µg L^−1^, respectively (*p*-value = 0.99), and UcCd = 0.57 ± 0.56 µg g^−1^ creatinine versus 0.68 ± 0.76, respectively (*p*-value = 0.73).

**Figure 4:**
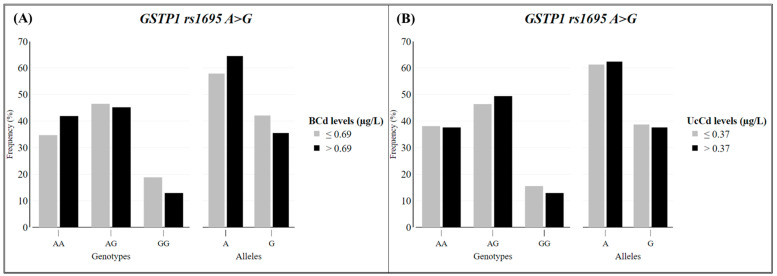
Distribution of Cd levels in blood (BCd) (**A**) and urine corrected by creatinine (UcCd) (**B**) samples according to *GSTP1* rs1695 A>G genotypes and alleles (*n* = 198 and *n* = 170, respectively). There were no significant differences between groups (*p*-values > 0.05, chi-squared test).

The circulating levels of Cd were categorized according to the median values, since they did not present normal distributions (Table 1 and Figure 4). The sociodemographic characteristics and the presence of NCDs were analyzed between the groups of Cd levels in blood and urine corrected by creatinine (Table 1). Individuals with respiratory disease had significantly higher blood Cd levels (>0.69 µg L^−1^) than those without this disease (OR = 2.42 and 95% CI = 1.21–5.01). There were no significant differences in other variables (sociodemographic and NCDs) between the two groups of Cd levels.

The rate of the successful genotyping of the *GSTP1* rs1695 A>G SNP was 97.8%. The frequencies of genotypes *AA*, *AG*, and *AG* from the studied population were 38.1%, 45.9%, and 16.0%, respectively, and were consistent with HWE. Considering only the allelic frequency, the variant *GSTP1* rs1695 G allele was present in 38.8% of the individuals. There were no significant differences (*p*-values > 0.05, chi-squared test) between circulating Cd levels, categorized by median values, and the distribution of the *GSTP1* rs1695 SNP (Figure 4) in the univariate analysis and after adjustment for confounding factors (age and smoking status). There were no significant differences in mean Cd levels (BCd, UCd, and UcCd) among the three genotypes (AA, AG, and GG) of *GSTP1* rs1695.

**Figure 5:**
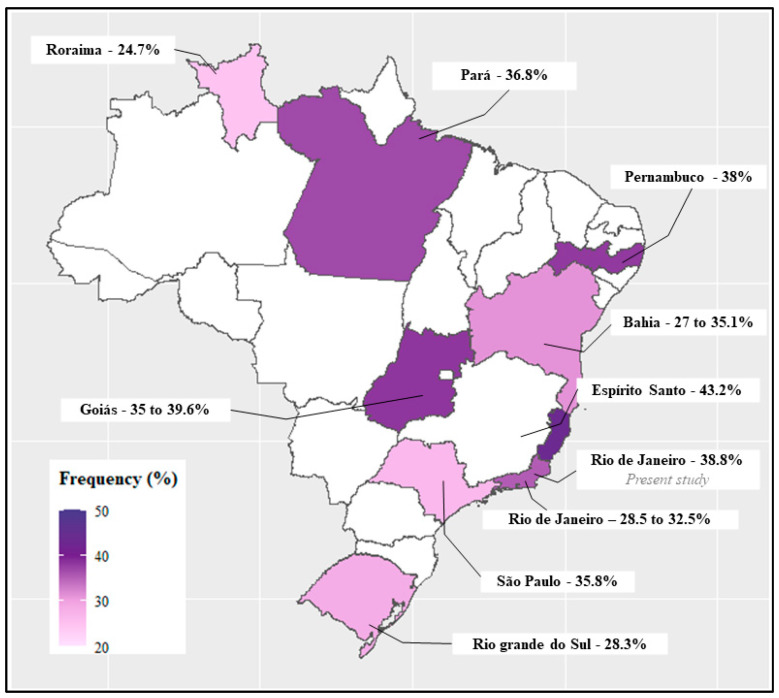
A map of Brazil showing the geographical regions of the country and the frequency of the *GSTP1* rs1695 *G*-variant allele according to literature data in the Brazilian population. White regions refer to states where the SNP has not yet been tested. There are no significant differences in the *GSTP1* rs1695 SNP frequency between the present study and previously published studies in other Brazilian regions (*p*-values > 0.05, qui-square test). References of each region: Rio de Janeiro [19,20], São Paulo [21], Rio Grande do Sul [13], Bahia [22,23], Roraima [24], Pernambuco [25], Espirito Santo [26], Goiás [27,28,29,30], and Pará [17].

The frequency of the G-variant allele of the *GSTP1* rs1695 SNP in the present study was described in comparison with previously published studies conducted in Brazil (Figure 5), and there were no statistical differences between the Brazilian Federal States (*p*-values > 0.05, qui-square test).

## 4. Discussion

The present study investigated Cd concentrations in samples from chronically exposed individuals living in a condominium built on an industrial waste site. Cd levels in urine are used as biomarkers of chronic exposure, reflecting the current body burden of the metal, while Cd in blood is a recent exposure biomarker [31]. Cd is widely known for its toxicity to mammals [4,5,6,7,8], and in this context, it is essential to consider that this population is at risk, since all the individuals had some level of Cd in their bodies. Among the evaluated subjects, the lowest levels were as follows: 0.28 µg L^−1^ in blood, 0.18 µg L^−1^ in urine, and 0.09 µg g^−1^ in urine corrected by creatinine. However, long-term health effects can still appear. The estimated half-life of Cd in blood ranges from 75 to 128 days [32], and to make matters worse, these individuals are constantly exposed to Cd because they live for about 15 years in an area where industrial waste has been dumped.

The level of Cd in the body can be increased by many variables, including both intrinsic and extrinsic factors [10,16,33]. For example, Cd concentrations in blood and urine tend to be higher in women than in men [10,34,35,36,37,38,39]. Women naturally have lower levels of iron in their bodies, and metabolic disorders related to iron loss are more significant in them. Therefore, a possible reason for this difference is the higher Cd absorption in low-iron stores [10]. Although there was no significant difference between the groups, in the present study, it was observed that more than 60% of the women had higher concentrations of Cd in their blood (BCd > 0.69 µg L^−1^) and corrected urine (UcCd > 0.37 µg g^−1^ creatinine) compared to men (36% and 39%, respectively). Smoking status is also associated with the presence of Cd in the body [10,16,33,40,41], as each cigarette contains approximately 1–2 µg of this metal, of which 25–35% is absorbed into the bloodstream [42]. In addition, exposure to secondhand smoke results in Cd concentrations in blood and urine that are 2 to 3 times higher than those of active smokers [43]. Despite the lack of statistical significance, in the current study, considering only individuals who smoke, 60% and 57% of them had high levels of Cd in their blood (BCd > 0.69 µg L^−1^) and urine (UcCd > 0.37 µg g^−1^ creatinine), respectively.

A comparison of the results obtained for BCd with the averages of studies, 0.10 µg L^−1^ [44] and 0.21 µg L^−1^ [45], both carried out in Brazil, in urban populations exposed to environmental Cd, shows values 7 and 3.5 times higher than those of these studies. Both established the same reference value for these populations, equal to 0.60 µg L^−1^ for BCd in adults over 18 years of age in the metropolitan region of the city of São Paulo, the largest city in Brazil [44,45]. In relation to this reference value, the average found in the current study is still 1.2 times higher. Compared to a study carried out on a Brazilian population exposed occupationally (BCd = 0.47 ± 0.31 µg L^−1^) [46], the Cd level in the blood of the residents of the Volta Redonda condominium is 1.5 times higher. In Brazil, the only existing legislation on human exposure to cadmium refers only to occupational exposure, which is extremely high (UcCd = 5 µg g^−1^ creatinine). Moreover, the Biological Exposure Index, which has clinical significance, highlights organic dysfunctions and adverse health effects and does not serve to prevent the effects caused by exposure [47].

Among genetic factors that can modulate Cd levels and consequently Cd toxicity, the *GSTP1* gene stands out, since it is a phase II detoxification enzyme and, therefore, is essential for the detoxification of xenobiotics, such as Cd [48,49]. The *GSTP1* rs1695 A>G SNP was associated with higher blood Cd levels (0.71 ± 0.08 µg L^−1^) in subjects carrying the variant genotype (*GSTP1* GG) in comparison to wild-type (*GSTP1* AA, Cd = 0.45 ± 0.02 µg L^−1^) or heterozygous (*GSTP1* AG, Cd = 0.45 ± 0.03 µg L^−1^) genotype carriers [11]. Similarly, an association between the *GSTP1* rs1695 SNP and high Cd levels (OR = 1.05, 95% CI = 1.01–1.13) was found in a case–control study assessing the risk of endometrial cancer [50]. The amino acid substitution (Ile105Val) in the presence of the *GSTP1* rs1695 313A>G SNP alters the catalytic activity of the enzyme, reducing the conjugation and excretion of substrates, justifying the accumulation of Cd in the body [11,14,51]. Possibly due to the limited number of individuals included in the present study, no significant differences were observed between *GSTP1* rs1695 genotypes and Cd levels in blood and urine. However, the SNP has a relatively high frequency in different regions of Brazil (Figure 5), and because of its biological function, it is a candidate SNP for assessing the genetic susceptibility of individuals more prone to accumulate it in the body and, therefore, to disease development.

The prevalence of NCDs in the studied population was higher than that found in the Brazilian population as a whole. According to Simões and collaborators in 2021 [52], cardiovascular disease had a prevalence of 5.3%, respiratory disease 1.6%, and renal disease 1.5%, versus 19%, 13%, and 2.5%, respectively, found in the present study. According to a study that evaluated the trends of BCd and UCd by pre-existing NCDs among adult participants of the U.S. National Health and Nutrition Examination Survey [10], people with chronic diseases were more likely to present higher Cd levels and were also at risk of presenting its adverse health effects. In the current study, higher blood Cd levels (>0.69 µg L^−1^) were associated (~2.5-fold) with respiratory disease, consistent with those previously described by Ganguly et al., 2018 [53]. Considering that environmental exposure to Cd plays a key role in the development of chronic diseases and that this metal is already considered as a probable human carcinogen [10], further studies are needed to evaluate the associations between Cd and disease risk.

The lack of significant associations between exposure and susceptibility biomarkers and variables evaluated in the present study may be related to the small sample size, which is the main limitation of this study. In addition, it was not possible to perform additional functional studies to provide evidence of toxicity. However, the findings using Cd biomarkers suggest that this study’s subjects had environmental exposure to the metal, and it is important to monitor these exposed residents before they develop more serious health outcomes. The construction of the condominium on a site contaminated by steel industry waste is a cause for concern and requires the continuous monitoring of its occupants to identify early at-risk individuals for intervention.

## 5. Conclusions

Our results highlight the vulnerable situation of the residents of the condominium built on a site contaminated by waste from the steel industry, due to the high levels of Cd and the increased prevalence of NCDs. In cases of chronic exposure, an analysis of Cd exposure levels is more informative/accurate in inferring possible toxic damage from the metal than an assessment of genetic susceptibility through an analysis of the *GSTP1* SNP. Therefore, it is crucial to identify those individuals who are more susceptible to Cd accumulation and consequently at higher risk of developing disease in order to provide immediate specific treatment.

## Figures and Tables

**Table 1:** Association between characteristics of studied population according to median Cd levels.

Characteristics	BCd ^a,b^ (µg L^−1^)			UcCd ^c,d^ (µg g^−1^)		
	≤0.69 (*n* = 101)	>0.69 (*n* = 97)	*p*-Value ^d^	≤0.37 (*n* = 84)	>0.37 (*n* = 86)	*p*-Value ^d^
**Sex**	**N (%)**			**N (%)**		
Women	59 (58.4)	62 (63.9)	0.5	51 (60.7)	52 (60.5)	1.0
Men	42 (41.6)	35 (36.1)		33 (39.3)	34 (39.5)	
**Age (years)**						
≤50	57 (56.4)	44 (45.4)	0.2	47 (56.0)	36 (41.9)	0.1
>50	44 (43.6)	53 (54.6)		37 (44.0)	50 (58.1)	
**Smoking status ^e^**						
Non-smoker	60 (76.9)	51 (64.6)	0.1	53 (75.7)	49 (66.2)	0.3
Smoker ^f^	18 (23.1)	28 (35.4)		17 (24.3)	25 (33.8)	
**Drinking status ^g^**						
Non-drinker	37 (46.2)	45 (57.0)	0.2	34 (47.9)	42 (56.0)	0.4
Drinker ^h^	43 (53.8)	34 (43.0)		37 (52.1)	33 (44.0)	
**Residence time (years) ^i^**						
≤17	56 (58.3)	47 (58.0)	1.0	46 (60.5)	43 (56.6)	0.7
>17	40 (41.7)	34 (42.0)		30 (39.5)	33 (43.4)	
**NCDs ^j^**						
Cardiovascular	34 (33.7)	31 (32.0)	0.9	34 (40.5)	23 (26.7)	0.1
Neurological	8 (7.9)	3 (3.1)	0.2	4 (4.8)	4 (4.7)	1.0
Renal	7 (6.9)	7 (7.2)	1.0	7 (8.3)	4 (4.7)	0.5
Respiratory	15 (14.9)	29 (29.9)	**0.02**	20 (23.8)	19 (22.1)	0.9
Neoplastic	5 (5.0)	1 (1.0)	0.2	5 (6.0)	1 (1.2)	0.2
**NCDs ^j^**						
No	49 (48.5)	43 (44.3)	0.6	36 (42.9)	44 (51.2)	0.4
Yes ^k^	52 (51.5)	54 (55.7)		48 (57.1)	42 (48.8)	

^a^ BCd levels in µg L^−1;^ ^b^ BCd: cadmium in blood; ^c^ UcCd levels in µg g^−1^ creatinine; ^d^ UcCd: cadmium in urine corrected by creatinine. ^e^ *p*-value obtained from the χ^2^ test (Pearson *p*-value) or Fisher’s exact test, as appropriate. ^f^ Missing information from 41 individuals (*n* = 157). ^g^ Includes former smokers or former drinkers. ^h^ Missing information from 39 individuals (*n* = 159). ^i^ Missing information from 18 individuals (*n* = 183). ^j^ NCDs: noncommunicable diseases. ^k^ One individual can present more than one noncommunicable disease.

## Data Availability

Data are contained within this article.

## References

[1] Pagliari B.G., Moreira M.D.F.R., Mannarino C.F., Santos G.B.D. (2021). Risk of Exposure to Metals in Soil Contaminated by Steel Industry Waste for a Population in Volta Redonda, RJ. Rev. Ambiente Água.

[2] Wu X., Cobbina S.J., Mao G., Xu H., Zhang Z., Yang L. (2016). A Review of Toxicity and Mechanisms of Individual and Mixtures of Heavy Metals in the Environment. Environ. Sci. Pollut. Res..

[3] Chen Y., Qu J., Sun S., Shi Q., Feng H., Zhang Y., Cao S. (2021). Health Risk Assessment of Total Exposure from Cadmium in South China. Chemosphere.

[4] Genchi G., Sinicropi M.S., Lauria G., Carocci A., Catalano A. (2020). The Effects of Cadmium Toxicity. Int. J. Environ. Res. Public Health.

[5] Jiang Y.-L., Fei J., Cao P., Zhang C., Tang M.-M., Cheng J.-Y., Zhao H., Fu L. (2022). Serum Cadmium Positively Correlates with Inflammatory Cytokines in Patients with Chronic Obstructive Pulmonary Disease. Environ. Toxicol..

[6] Marzec J.M., Nadadur S.S. (2022). Inflammation Resolution in Environmental Pulmonary Health and Morbidity. Toxicol. Appl. Pharmacol..

[7] Unsal V., Dalkiran T., Çiçek M., Kölükçü E. (2020). The Role of Natural Antioxidants Against Reactive Oxygen Species Produced by Cadmium Toxicity: A Review. Adv. Pharm. Bull..

[8] Rana M.N., Tangpong J., Rahman M.M. (2018). Toxicodynamics of Lead, Cadmium, Mercury and Arsenic- Induced Kidney Toxicity and Treatment Strategy: A Mini Review. Toxicol. Rep..

[9] Riederer A.M., Belova A., George B.J., Anastas P.T. (2013). Urinary Cadmium in the 1999–2008 U.S. National Health and Nutrition Examination Survey (NHANES). Environ. Sci. Technol..

[10] Yang J., Lo K., Yang A. (2022). Trends in Urinary and Blood Cadmium Levels in U.S. Adults with or without Comorbidities, 1999–2018. Nutrients.

[11] Khansakorn N., Wongwit W., Tharnpoophasiam P., Hengprasith B., Suwannathon L., Chanprasertyothin S., Sura T., Kaojarern S., Sritara P., Sirivarasai J. (2012). Genetic Variations of Glutathione S-Transferase Influence on Blood Cadmium Concentration. J. Toxicol..

[12] Rossini A., Rapozo D.C.M., Amorim L.M.F., Macedo J.M.B., Medina R., Neto J.F.N., Gallo C.V.M., Pinto L.F.R. (2002). Frequencies of GSTM1, GSTT1, and GSTP1 Polymorphisms in a Brazilian Population. Genet. Mol. Res..

[13] Da Silva J., Moraes C.R., Heuser V.D., Andrade V.M., Silva F.R., Kvitko K., Emmel V., Rohr P., Bordin D.L., Andreazza A.C. (2008). Evaluation of Genetic Damage in a Brazilian Population Occupationally Exposed to Pesticides and Its Correlation with Polymorphisms in Metabolizing Genes. Mutagenesis.

[14] Strange R.C., Jones P.W., Fryer A.A. (2000). Glutathione S-Transferase: Genetics and Role in Toxicology. Toxicol. Lett..

[15] Baghaei A., Behjati M., Karimian A. (2022). Association Analysis of GSTP1-Rs1695 Polymorphism with the Risk of Oral Cancer: A Literature Review, an Updated Meta- Analysis, and a Structural Assessment. Asian Pac. J. Cancer Prev..

[16] Perini J.A., Silva M.C.D., Correa L.V., Silva Y.M., Borges R.M., Moreira M.D.F.R. (2022). Chronic Cadmium Exposure and Genetic Polymorphisms of MMP-2 and MMP-9 in a Population Exposed to Steel Slag in the State of Rio de Janeiro, Brazil: A Cross-Sectional Study. Int. J. Environ. Res. Public Health.

[17] Silva M.C.D., Oliveira R.A.A.D., Vasconcellos A.C.S.D., Rebouças B.H., Pinto B.D., Lima M.D.O., Jesus I.M.D., Machado D.E., Hacon S.S., Basta P.C. (2023). Chronic Mercury Exposure and GSTP1 Polymorphism in Munduruku Indigenous from Brazilian Amazon. Toxics.

[18] Da Silva M.C., Basta P.C., Hofer C.B., de Oliveira M.A.F., Kempton J.W., de Oliveira R.A.A., de Vasconcellos A.C.S., Perini J.A. (2024). The GSTP1 Rs1695 Polymorphism Is Associated with Mercury Levels and Neurodevelopmental Delay in Indigenous Munduruku Children from the Brazilian Amazon. Toxics.

[19] Rossini A., Rapozo D.C.M., Soares Lima S.C., Guimarães D.P., Ferreira M.A., Teixeira R., Kruel C.D.P., Barros S.G.S., Andreollo N.A., Acatauassú R. (2007). Polymorphisms of GSTP1 and GSTT1, but Not of CYP2A6, CYP2E1 or GSTM1, Modify the Risk for Esophageal Cancer in a Western Population. Carcinogenesis.

[20] Hatagima A., Costa E.C.B., Marques C.F.S., Koifman R.J., Boffetta P., Koifman S. (2008). Glutathione S-Transferase Polymorphisms and Oral Cancer: A Case-Control Study in Rio de Janeiro, Brazil. Oral. Oncol..

[21] Honma H.N., De Capitani E.M., Perroud M.W., Barbeiro A.S., Toro I.F.C., Costa D.B., Lima C.S.P., Zambon L. (2008). Influence of P53 Codon 72 Exon 4, GSTM1, GSTT1 and GSTP1*B Polymorphisms in Lung Cancer Risk in a Brazilian Population. Lung Cancer.

[22] Magno L.A.V., Talbot J., Talbot T., Borges Santos A.M., Souza R.P., Marin L.J., Moreli M.L., De Melo P.R.S., Corrêa R.X., Rios Santos F. (2009). Glutathione S-Transferase Variants in a Brazilian Population. Pharmacology.

[23] Rocha A.V., Talbot T., Magalhães da Silva T., Almeida M.C., Menezes C.A., Di Pietro G., Rios-Santos F. (2011). Is the GSTM1 Null Polymorphism a Risk Factor in Primary Open Angle Glaucoma?. Mol. Vis..

[24] Lima Junior M.M.D., Reis L.O., Ferreira U., Cardoso U.O., Barbieri R.B., Mendonça G.B.D., Ward L.S. (2015). Unraveling Brazilian Indian Population Prostate Good Health: Clinical, Anthropometric and Genetic Features. Int. Braz. J. Urol..

[25] Chagas B.S., Gurgel A.P.A.D., Júnior S.S.L.P., Lima R.C.P., Cordeiro M.N., Moura R.R., Coelho A.V.C., Nascimento K.C.G., Neto J.C.S., Crovella S. (2017). Research Article Synergic Effect of Oral Contraceptives, GSTP1 Polymorphisms, and High-Risk HPV Infection in Development of Cervical Lesions. Genet. Mol. Res..

[26] Dos Santos E.V.W., Alves L.N.R., Louro I.D. (2017). Steroid Metabolism Gene Polymorphisms and Their Implications on Breast and Ovarian Cancer Prognosis. Genet. Mol. Res..

[27] de Lima R.M., Dos Anjos L.R.B., Alves T.B., Coelho A.S.G., Pedrino G.R., da Silva Santos R., da Silva Cruz A.H., da Silva Reis A.A. (2018). Do GST Polymorphisms Influence in the Pathogenesis of Diabetic Nephropathy?. Mol. Cell Endocrinol..

[28] Oliveira De Araújo Melo C., Cidália Vieira T., Duarte Gigonzac M.A., Soares Fortes J., Moreira Duarte S.S., Da Cruz A.D., Silva D.D.M.E. (2020). Evaluation of Polymorphisms in Repair and Detoxification Genes in Alcohol Drinkers and Non-drinkers Using Capillary Electrophoresis. Electrophoresis.

[29] Barros J.B.d.S., Santos K.d.F., Azevedo R.M., de Oliveira R.P.D., Leobas A.C.D., Bento D.d.C.P., Santos R.d.S., Reis A.A.d.S. (2021). No Association of GSTP1 Rs1695 Polymorphism with Amyotrophic Lateral Sclerosis: A Case-Control Study in the Brazilian Population. PLoS ONE.

[30] de Sousa Barros J.B., de Faria Santos K., da Cruz Pereira Bento D., do Prado Assunção L., da Silva Santos R., da Silva Reis A.A. (2022). Influence of GSTP1 Rs1695 Polymorphism on Survival in Male Patients’ Amyotrophic Lateral Sclerosis: A Genetic Association Study in Brazilian Population. Mol. Biol. Rep..

[31] Sigel R.K.O., Skilandat M., Sigel A., Operschall B.P., Sigel H., Sigel A., Sigel H., Sigel R.K. (2013). Complex Formation of Cadmium with Sugar Residues, Nucleobases, Phosphates, Nucleotides, and Nucleic Acids. Cadmium: From Toxicity to Essentiality.

[32] Bernhoft R.A. (2013). Cadmium Toxicity and Treatment. Sci. World J..

[33] Kim J., Song H., Lee J., Kim Y.J., Chung H.S., Yu J.M., Jang G., Park R., Chung W., Oh C.-M. (2023). Smoking and Passive Smoking Increases Mortality through Mediation Effect of Cadmium Exposure in the United States. Sci. Rep..

[34] Rooney J.P.K., Michalke B., Geoghegan G., Heverin M., Bose-O’Reilly S., Hardiman O., Rakete S. (2022). Urine Concentrations of Selected Trace Metals in a Cohort of Irish Adults. Env. Sci. Pollut. Res..

[35] Moon J.-Y., Eom S.-Y., Seo J.-W., Lee J.-E., Choi B.-S., Kim H., Hong Y.-S., Chang J.Y., Jeon M.-J., Park W.-J. (2021). Effects of Exposure to Lead and Cadmium on Health of Inhabitants of Abandoned Metal Mine Area in Korea. Arch. Env. Contam. Toxicol..

[36] Park E., Kim J., Kim B., Park E.Y. (2021). Association between Environmental Exposure to Cadmium and Risk of Suspected Non-Alcoholic Fatty Liver Disease. Chemosphere.

[37] Satarug S., Gobe G.C., Ujjin P., Vesey D.A. (2020). A Comparison of the Nephrotoxicity of Low Doses of Cadmium and Lead. Toxics.

[38] Eom S.-Y., Seo M.-N., Lee Y.-S., Park K.-S., Hong Y.-S., Sohn S.-J., Kim Y.-D., Choi B.-S., Lim J.-A., Kwon H.-J. (2017). Low-Level Environmental Cadmium Exposure Induces Kidney Tubule Damage in the General Population of Korean Adults. Arch. Env. Contam. Toxicol..

[39] Garner R.E., Levallois P. (2017). Associations between Cadmium Levels in Blood and Urine, Blood Pressure and Hypertension among Canadian Adults. Environ. Res..

[40] Ratelle M., Li X., Laird B.D. (2018). Cadmium Exposure in First Nations Communities of the Northwest Territories, Canada: Smoking Is a Greater Contributor than Consumption of Cadmium-Accumulating Organ Meats. Env. Sci. Process Impacts.

[41] Shakeri M.T., Nezami H., Nakhaee S., Aaseth J., Mehrpour O. (2021). Assessing Heavy Metal Burden Among Cigarette Smokers and Non-Smoking Individuals in Iran: Cluster Analysis and Principal Component Analysis. Biol. Trace Elem. Res..

[42] Järup L., Åkesson A. (2009). Current Status of Cadmium as an Environmental Health Problem. Toxicol. Appl. Pharmacol..

[43] Garner R., Levallois P. (2016). Cadmium Levels and Sources of Exposure among Canadian Adults. Health Rep..

[44] Kuno R., Roquetti M.H., Becker K., Seiwert M., Gouveia N. (2013). Reference Values for Lead, Cadmium and Mercury in the Blood of Adults from the Metropolitan Area of Sao Paulo, Brazil. Int. J. Hyg. Environ. Health.

[45] Kira C.S., Sakuma A.M., De Capitani E.M., De Freitas C.U., Cardoso M.R.A., Gouveia N. (2016). Associated Factors for Higher Lead and Cadmium Blood Levels, and Reference Values Derived from General Population of São Paulo, Brazil. Sci. Total Environ..

[46] Ferron M.M., Kuno R., Campos A.E.M.D., Castro F.J.V.D., Gouveia N. (2020). Cadmium, Lead and Mercury in the Blood of Workers from Recycling Sorting Facilities in São Paulo, Brazil. Cad. Saúde Pública.

[47] NR 7—Programa de Controle Médico de Saúde Ocupacional—PCMSO Atualizada pela Portaria MPT nº 567, em 10/03/2022. https://www.gov.br/trabalho-e-emprego/pt-br/acesso-a-informacao/participacao-social/conselhos-e-orgaos-colegiados/comissao-tripartite-partitaria-permanente/arquivos/normas-regulamentadoras/nr-07-atualizada-2022-1.pdf.

[48] Zhang H., Reynolds M. (2019). Cadmium Exposure in Living Organisms: A Short Review. Sci. Total Environ..

[49] Lei X., Du L., Yu W., Wang Y., Ma N., Qu B. (2021). GSTP1 as a Novel Target in Radiation Induced Lung Injury. J. Transl. Med..

[50] Michalczyk K., Kapczuk P., Witczak G., Bosiacki M., Kurzawski M., Chlubek D., Cymbaluk-Płoska A. (2022). The Associations between Metalloestrogens, GSTP1, and SLC11A2 Polymorphism and the Risk of Endometrial Cancer. Nutrients.

[51] Dusinská M., Ficek A., Horská A., Raslová K., Petrovská H., Vallová B., Drlicková M., Wood S.G., Stupáková A., Gasparovic J. (2001). Glutathione S-Transferase Polymorphisms Influence the Level of Oxidative DNA Damage and Antioxidant Protection in Humans. Mutat. Res..

[52] Simões T.C., Meira K.C., Santos J.D., Câmara D.C.P. (2021). Prevalências de doenças crônicas e acesso aos serviços de saúde no Brasil: Evidências de três inquéritos domiciliares. Ciênc. Saúde Coletiva.

[53] Ganguly K., Levänen B., Palmberg L., Åkesson A., Lindén A. (2018). Cadmium in Tobacco Smokers: A Neglected Link to Lung Disease?. Eur. Respir. Rev..

